# PHYLOViZ: phylogenetic inference and data visualization for sequence based typing methods

**DOI:** 10.1186/1471-2105-13-87

**Published:** 2012-05-08

**Authors:** Alexandre P Francisco, Ctia Vaz, Pedro T Monteiro, José Melo-Cristino, Mário Ramirez, Joo A Carrio

**Affiliations:** 1 KDBIO, INESC-ID, , R. Alves Redol 9, 1000-029 Lisboa, Portugal; 2 CSE Dept, IST, Tech Univ of Lisbon, Av. Rovisco Pais 1, 1049-001 Lisboa, Portugal; 3 DEETC, ISEL, Poly Inst of Lisbon, R. Cons. Emdio Navarro 1, 1959-007 Lisboa, Portugal; 4 Inst de Microbiologia, Inst de Medicina Molecular, Fac de Medicina, Univ of Lisbon, Av. Prof. Egas Moniz, 1649-028 Lisboa, Portugal; 5 Instituto Gulbenkian de Ciência, 2781-901 Oeiras, Portugal

## Abstract

**Background:**

With the decrease of DNA sequencing costs, sequence-based typing methods are rapidly becoming the gold standard for epidemiological surveillance. These methods provide reproducible and comparable results needed for a global scale bacterial population analysis, while retaining their usefulness for local epidemiological surveys. Online databases that collect the generated allelic profiles and associated epidemiological data are available but this wealth of data remains underused and are frequently poorly annotated since no user-friendly tool exists to analyze and explore it.

**Results:**

PHYLOViZ is platform independent Java software that allows the integrated analysis of sequence-based typing methods, including SNP data generated from whole genome sequence approaches, and associated epidemiological data. goeBURST and its Minimum Spanning Tree expansion are used for visualizing the possible evolutionary relationships between isolates. The results can be displayed as an annotated graph overlaying the query results of any other epidemiological data available.

**Conclusions:**

PHYLOViZ is a user-friendly software that allows the combined analysis of multiple data sources for microbial epidemiological and population studies. It is freely available at http://www.phyloviz.net.

## Background

Microbial typing methods are fundamental tools for epidemiological studies and for the study of bacterial populations. These techniques allow the characterization of bacteria at the strain level providing researchers with important information for the surveillance of infectious diseases, outbreak investigation, pathogenesis studies, natural history of an infection and bacterial population genetics. The recent advances in sequencing technologies and the resulting decrease in costs, are promoting sequence based typing methods over traditional mole- cular methodologies, due to their reproducibility and portability of results, which allows for straightforward inter-laboratory comparison of microbial strains.

Multilocus Sequence Typing (MLST) [1] and Multilocus Variable Number of Tandem Repeats Analysis (MLVA) [2] are examples of such methods that are available for a multitude of bacterial species and are being used globally in epidemiological microbial typing and bacterial population studies [3]. Over seventy different microorganisms have public MLST databases available at five different online locations, namely MLST.net [4], PubMLST [5], Institut Pasteur MLST Databases [6], EcMLST [7] and MLST Databases at the ERI [8]. With the advent of Next Generation Sequencing (NGS), Single Nucleotide Polymorphism (SNP) analysis of entire bacterial genomes is being increasingly used [9-12] and may eventually in the future replace MLST as the typing method of choice in global epidemiological studies.

These advances created the need for algorithms and tools to make sense of this wealth of data in the context of epidemiology, population genetics and evolution. Existing free software tools for data analysis and visualization of microbial typing data are focused on MLST analysis, including the algorithms START [13], eBURST [14] and goeBURST [15]. However, these tools lack the capacity to integrate epidemiological data, which is crucial for the correct inference and analysis of relationships between strains. Other well known software tools, provide the ability for network visualization and data integration, usually of generic use, such as Gephi [16] and Tulip [17] or more focused on biological networks such as regulatory, protein interaction or metabolic networks, as is the case of Cytoscape [18]. Neither these nor any other current tools for network visualization [19] are specifically directed towards population or evolutionary analysis and all depend on already defined trees or networks, lacking the ability to infer them directly from data origi- nating from sequence-based typing methods. Moreover, given their focus on general use, their configuration and customization for epidemiological data analysis requires expertise on graph theory and network mining and frequently calls for programming skills, so that their use for this purpose is restricted only to a few advanced users.

In order to cover the existing gap between the potential users and the tools available for data analysis and visualization of sequence-based typing methods and associated epidemiological and population data, we developed PHYLOViZ. The most similar tool currently available is the eBURST software (available at http://eburst.mlst.net) that offers a heuristic implementation of the BURST algorithm, a radial static display of the generated trees and was designed to analyze solely MLST data. PHYLOViZ was designed with an open architecture, building upon the goeBURST implementation (available at http://goeburst.phyloviz.net). It allows the expansion of the types of data that can be analyzed as well as of the algorithms and metrics used to analyze it. Its main goal is to integrate and to display multiple sources of information. In the current implementation, both allelic data from MLST, as well as from MLVA and the actual nucleic acid sequence data are supported for analysis by PHYLOViZ. The software is also able to integrate ancillary information that may consist of clinical or patient demographic data or additional isolate information such as the results of other phenotypic or genotypic typing methods. PHYLOViZ and the implemented algorithms are capable of handling large data sets, a growing requirement in the microbial typing field, with the constantly increasing developments in sequencing technology. PHYLOViZ’s design adhered to the Visual Information Seeking Mantra of “overview first, zoom and filter, then details on demand” [20], aiming to allow users to both find a narrow set of items in a large collection and display the relationships between them, but also to develop an understanding of unexpected patterns within the collection by empowering the user with query and display tools. The capacity to dynamically explore the data is what allows an effective analysis of large data sets.

**Figure 1:**
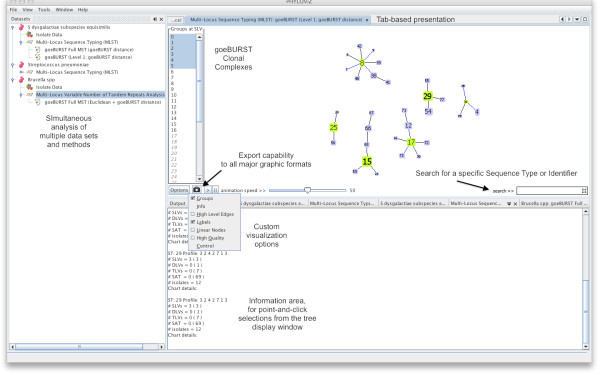
**PHYLOViZ basic interface features**. Illustration of the basic interface features of PHYLOViZ.

## Implementation

### Overview

PHYLOViZ allows users to manipulate multiple data sets of sequence-based typing data for analysis, which are presented within a window with a tree based navigation menu. Each data set is described by a file containing typing data, i.e., allelic profiles or nucleotide sequences generated by a sequence based typing method such as MLST, MLVA or SNP data. Moreover, a file containing ancillary data, sometimes also refered to as metadata, such as epidemiological, demographic information or any other relevant data may also be added and linked to the allelic profile data, representing information available for different isolates. Both typing data profiles and isolate data can be visualized in a table or a tree view. These views allow the user to query and filter the data using regular expressions or by using a simple point and click interface as described in detail below. Figure 1 displays the basic interface functionalities of the PHYLOViZ interface.

Once the data files are loaded, the user can infer probable patterns of evolutionary descent between allelic profiles using the goeBURST algorithm or its expansion to generalized Minimum Spanning Tree (MST) like structures using different distance metrics. The unrooted trees resulting from the algorithms are displayed using a force-directed layout, where they can be interactively visualized, freely manipulated by the user to explore the existing links and interrogated, either through clicking on the nodes or links of interest to display additional information or by searching the graph for specific nodes.

Users can then combine or query specific characteristics of either typing or ancillary data and overlay the results onto the graphical display. Queries can be easily performed in a tree menu view, by selecting character values to be displayed onto the tree using selection boxes. A regular expression composition box is also provided to allow more complex queries. The resulting annotated trees can then be exported in a variety of common graphic formats, including both vector and bitmap formats. A complete tutorial for PHYLOViZ and a description of its features is available at http://www.phyloviz.net/wiki/tutorial.

### Architecture

The software architecture is based on the NetBeans Platform (available at http://platform.netbeans.org), which includes a plugin system. Users can develop and use new plugins according to their requirements and make them available for the community through their own website or by depositing them at the PHYLOViZ repository.

It is our objective to keep PHYLOViZ as modular and extensible as possible, allowing a seamless integration of new data analysis methods and visualization features. PHYLOViZ is written in Java and is compatible with any operating system running Java 1.6 or higher. The software uses several well-known open source libraries for data export and visualization, notably the Prefuse toolkit for interactive information visualization (available at http://prefuse.org) and the FreeHEP Java libraries for image exporting (available at http://java.freehep.org). The code for PHYLOViZ core and most plugins is available under the GNU General Public License (GPL) version 3 or any later version, with a Classpath Exception. The Classpath Exception means that the copyright holders of a library give you permission to link this library with independent modules to produce an executable, regardless of the license terms of these independent modules. More complete information on the license is provided at http://www.phyloviz.net/wiki/download/. Both the source code and binary versions for major operating systems, as well as the documentation are available at http://www.phyloviz.net.

### Algorithmics

The current version of PHYLOViZ supports the inference of patterns of evolutionary descent using the goeBURST algorithm [15] and its extension to a full MST-like approach.

The first improvement over the original implementation of the goeBURST algorithm is that PHYLOViZ allows any possible distance for linkage comparison. The user can choose among available distances, or add new distances by implementing the interface AbstractDistance (see Data Model section) and providing a new plugin as described in Supplemental Material. In order to allow such extensibility, we have identified basic distance requisites. Thus, any implemented distance should provide a way to compare two links (i.e., a link comparator), and it should also compute a level for each link. Note that only a link comparator is required for most MST like approaches and, in particular, for goeBURST. The level is only relevant for link filtering, as is traditionally done for filtering links at different number of locus variants in the case of MLST data analysis. When link levels are not applicable for a given distance measure, we can define the level as being equal for all links.

Secondly, our implementation extends the goeBURST rules to any number of variable loci in order to create a single tree. This approach uses the goeBURST rules to identify, out of all the possible MST-like trees, the one that is concordant with the evolutionary model supporting the rules, guaranteeing a unique and consistent solution. The MST analysis allows the user to dynamically visualize the groups formed at any linkage level, including those formed at single-locus variant (SLV) level (the standard result of goeBURST). The information of the group in which each isolate is classified, for each linkage level, can be exported to the ancillary data file and combined with existing data for later analysis.

The capacity to create groups at any linkage level is particularly useful to analyze MLVA and SNP typing data, since schemata can have much larger number of loci than traditional MLST schemes, frequently relying only on seven loci. The higher number of loci could be expected to yield a larger number of differences between individuals, motivating the exploration of higher linkage levels to generate useful trees translating evolutionary hypotheses.

Note that by allowing any possible distance for link comparison and thereby fully generalizing link comparison, any problem of tree construction becomes a graphical matroid instance [15,21], that can be solved by well known algorithms, such as the Borǔvka algorithm [22], the Kruskal algorithm [23] or the Prim algorithm [24]. For goeBURST, we enumerated explicitly all possible links, because we computed tie statistics for them, and we built the tree using the Kruskal algorithm. However, for the full MST computation, we are now using the Prim algorithm. The main reason for this is to avoid enumerating explicitly all links, the number of which, in the generalized version, grows quadratically with the number of nodes. The Prim algorithm only requires us to generate links implicitly, involving at most to keep in memory as many links as the number of nodes and, thus, making the approach more scalable for large data sets. Moreover, since we may have dense graphs, we also implemented a more efficient priority queue for Prim’s algorithm, based on rank relaxed heaps as proposed by Driscoll et al. [25]. These improvements are part of the PHYLOViZ Algorithms plugin. The visualization of the full MST was also optimized to support large data sets, with thousands of nodes. This was made possible by incrementally adding nodes to the force directed display following a Breadth First Search approach starting from the node with greater number of links. This lead to increased optimization speed and lowered the memory requirements for the MST display.

### Creating new plugins

The PHYLOViZ plugin system allows new algorithms and visualization tools to be easily added. The implementation of these features must also use the NetBeans Platform respecting the documentation regarding the creation of new modules available at http://netbeans.org/kb/trails/platform.html.

In order to have access to the visualization and querying capabilities of PHYLOViZ, the developer must add the intended PHYLOViZ plugins as library dependencies of the new plugin. Extensive documentation, describing the data model and algorithms API, is available on the website and in the Data Model section. A plugin implementation example is provided in Supplemental Material.

The graphical user interface for the incorporation of newly developed plugins is found in the Plugins entry of the Tools menu. The user has the option of adding a plugin stored locally, or through the use of a remote Update Center. The latter is periodically checked for new versions of the installed plugins and the user notified in case of the availability of updates. The update center can also be used to install new publicly available plugins from within PHYLOViZ.

### Data model

**Figure 2:**
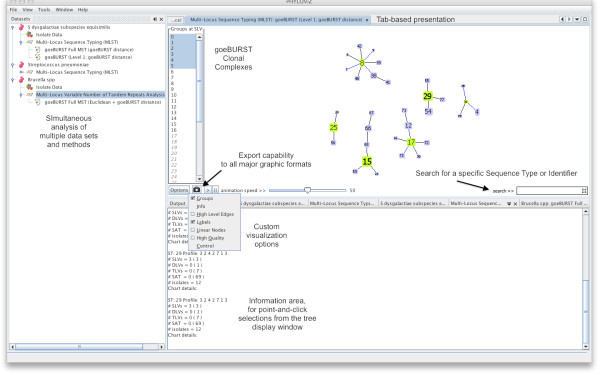
**PHYLOViZ data model**. UML diagram of the PHYLOViZ core data model.

The underlying data model of PHYLOViZ was designed to provide a suitable and flexible enough abstraction to represent data from different typing methods, and to provide an unified interface for developing different algorithms and tools for data analysis. The basic unit is the DataSet, which aggregates typing data and isolate ancillary data, as depicted in Figure 2. Both typing and ancillary data are collections of tuples. Each data tuple can be seen as a vector, named DataItem in the data model. By using such abstraction, we can implement data viewers and filters for both types of data at once, as we did in PHYLOViZ Table/Tree Viewer plugin and in PHYLOViZ Category plugin. Let us now consider how typing data and ancillary are represented and distinguished. Ancillary data is represented as a tuple of values for each isolate and, thus, in the data model, we have Isolate and Population, i.e., a collection of Isolates. One of the isolate tuple entries is distinguished as the foreign key that allows relating ancillary data to typing data. Similarly, typing data is represen- ted as a collection of identified tuples, known in the data model as TypingData and Profiles. These types have different APIs when compared with Population and Isolate, and in particular they provide all the frame- work required for algorithm implementation. Although TypingData is just a container that is used to represent collections of Profiles, a Profile is an interface that must be extended by each implementation that makes available a new kind of typing data. In the current version of the software, the Profile interface is implemented by the PHYLOViZ MLST, PHYLOViZ MLVA, PHYLOViZ SNP and PHYLOViZ Aligned Sequences plugins, allowing the input of different data files. This data model and several other helper classes are implemented in the PHYLOViZ Core.

We also provide a common API and utilities for algorithm implementation in the plugin PHYLOViZ Algorithms. The most relevant types are the AlgorithmProvider and the AbstractDistance. The first one is an interface that should be implemented by any algorithm so that PHYLOViZ can recognize it at runtime. The second one, AbstractDistance, is also an interface that should be implemented whenever we want to extend algorithms based on link comparison with new distances, as is the case of the MST and goeBURST algorithms. The documentation of this plugin, as well as the documentation of the full data model, is available online for users interested in developing new plugins.

**Figure 3:**
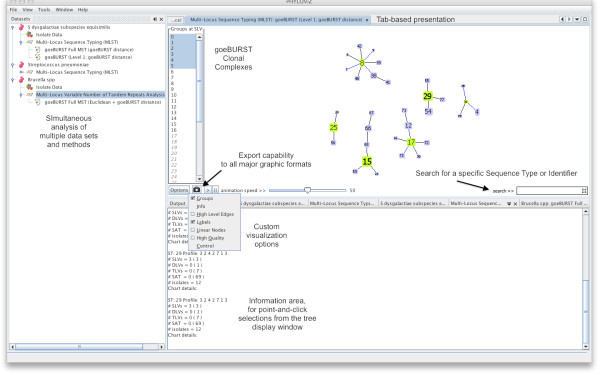
**Largest Clonal Complex defined by goeBURST for Streptococcus pneumoniae**. Largest Clonal Complex defined by goeBURST for the *Streptococcus pneumoniae* data set. It contains 986 unique Sequence Types (STs) for a total of 2260 isolates in the database.

## Results and Discussion

PHYLOViZ aims to be an efficient and user-friendly software, providing an interface where users can analyze and visualize their data, and export the results in several commonly used graphic formats.

To achieve these goals, it allows users to create data sets by loading the data from tab separated files for any kind of allelic profile data (be it MLST, MLVA or SNP data, i.e. Typing Data), that can be easily exported from Excel worksheets, or FASTA formatted files for sequence data, a common format easily obtained from any sequence analysis editor software. Nevertheless, as previously pointed out [15], the use of sequence information to infer relatedness between microbial isolates can be confounded by recombination that may introduce multiple nucleotide changes in a single event. On the other hand for microbial species where recombination is infrequent or for chromosomic regions where recombination is rare, this may be an approach that can be pursued. The users can also use the available plugins to download data directly from public databases that provide their data through a Web service interface such as PubMLST.org [5] or as simple flat files. This flexibility, coupled with the ability to use the goeBURST algorithm and its expansion to a MST, allows the user to load multiple data sets simultaneously for the analysis, visual exploration and comparison of multiple typing results for the same isolate collection. Any available additional ancillary clinical or typing data can be similarly easily exported from Excel worksheets or from other database software, or directly retrieved from PubMLST.org from within PHYLOViZ (see below).

Also, by applying the same analysis algorithms to different typing techniques, namely MLST, MLVA or SNP typing, PHYLOViZ provides researchers with a more direct comparison of the different typing methodologies results, since using the same underlying evolutionary models and assumptions avoids possible artifacts or differences resulting from using multiple data analysis techniques for each typing method and then comparing the results. For the analysis of MLVA data, an alternative distance based on goeBURST is also made available in the software. In this implementation, the algorithm still creates trees only linking SLVs, however instead of simply using the goeBURST rules to decide which links should be drawn, it starts by considering the euclidean distance between two profiles. This distance is calculated as the sum of the differences across all loci in the MLVA profile. For instance in a two locus scheme the difference between profiles 1-3 and 1-1 would be calculated as 2 in this metric whereas the profiles would be SLVs in the classic MLST distance. This assumes that the genetic distance between two MLVA profiles is proportional to the difference in the number of repeats at each locus. This is in accordance with models of accumulation of tandem repeats in each loci [26-28], and for some data sets could create a more realistic and meaningful representation than using only the goeBURST rules.

The ability to analyze data sets comprising thousands of isolates, with thousands of target loci, also allows PHYLOViZ to efficiently handle the large volumes of data generated by NGS when applied to molecular epidemiology. This, coupled with its ability for data integration with a simple query system and dynamic visualization capabilities provide a user friendly platform aimed at non-bioinformaticians such as clinical microbiologists, epidemiologists and infection control specialists.

### Phyloviz usage example

To demonstrate a typical usage of PHYLOViZ for exploring sequence-based typing methods databases and related epidemiological data, we show the analysis of the MLST database from *Streptococcus pneumoniae* retrieved from https://spneumoniae.mlst.net. This data set (retrieved in 28 October 2011) contains 7146 unique sequence types and information on 15329 isolates. The available data on penicilin susceptibility was curated and recoded for R (resistant), I (intermediate susceptibility) and S (susceptible) based on the Clinical and Laboratory Standards Institute breakpoints for oral penicillin [29] and saved on a tab delimited file. In subsequent analysis isolates classified as R or I are collectively referred to as non-susceptible. A new data set was created on PHYLOViZ were 7146 unique STs and respective allelic profiles were used as Typing Data and the penicillin resistance information was used as Isolate Data. The goeBURST algorithm [15] was computed at SLV level for the data set dividing it in 506 groups of two or more STs and 1514 singletons (STs unrelated to any other in the collection at SLV level). The largest clonal complex (CC), currently having 986 unique STs and representing 2260 isolates is presented in Figure 3.

**Figure 4:**
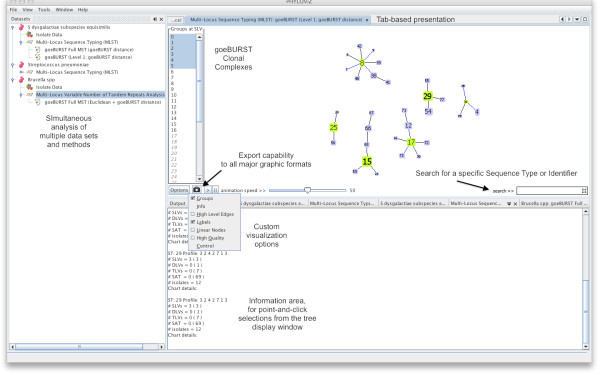
**Largest Clonal Complex defined by goeBURST for Streptococcus pneumoniae colored by Penicilin susceptibility**. Largest Clonal Complex defined by goeBURST for the *Streptococcus pneumoniae* data set colored by Penicilin susceptibility :Susceptible (Green) *MIC*≤0.064 mg/L; Intermediate (Orange) 0.09*mg*/*L*≤*MIC*≤1 mg/L; Resistant (Red) *MIC*>1 mg/L .The area in the inset will be further analyzed and described in Figure 5

By selecting the penicillin resistance column in the Isolate Data, and selecting View in the interface, the user can instantly display how the penicillin resistance varies throughout the whole data set. Figure 4 illustrates the overall representation of penicillin resistance for the largest CC. We can see that in the whole CC, penicillin non-susceptibility is concentrated in some areas of the whole tree.

**Figure 5:**
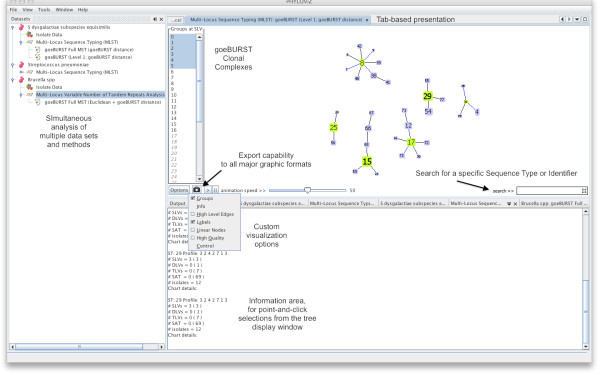
**ST 156 and ST 162 subgroups on the largest CC in Streptococcus pneumoniae**. ST 156 and ST 162 subgroups on the largest CC in *Streptococcus pneumoniae*. A) The colors represent Penicillin susceptibility : Susceptible (Green) *MIC*≤0.064 mg/L; Intermediate (Orange) 0.09*mg*/*L*≤*MIC*≤1 mg/L; Resistant (Red) *MIC*>1 mg/L . B) The colors represent the different *ddl* alelles. High frequency alleles displayed in the figure: *ddl* 14 - blue, *ddl* 1 - dark purple, *ddl* 90 - pink, *ddl* 119 - light purple - 119. C) Representation of the unique *ddl* alleles that are present in SLVs of ST 156 and of ST 162. The colors are arbitrarily assigned.

Focusing on one of those areas we can explore the graph locally. In Figure 5A we focused on the ST156 and ST162 subgroups. In *Streptococcus pneumoniae*, the major determinants of penicillin resistance are alterations of membrane proteins, known as Penicillin Binding Proteins (PBPs) [30]. PBPs are involved in the synthesis of peptidoglycan, the major component of the bacterial cell walls, and become inactivated when bound to penicillin. Some alterations in the PBP genes cause amino acid changes that impair the binding of beta-lactams antibiotics, leading to decreased susceptibility to penicillin [30]. In *S. pneumoniae*, the remodelling of the PBPs leading to resistance is frequently driven by recombination with exogenous DNA, probably through transformation [30]. It was shown that *ddl*, one of the genes used in the *S. pneumoniae* MLST scheme, shows variation due to a hitchhiking effect driven by its proximity on the chromosome to the gene encoding PBP2a involved in penicillin resistance [31]. The incorporation of DNA fragments encoding both genes from closely related streptococci was shown to affect the variability at both loci and could to also contribute to this effect. This can be easily visualized by displaying the *ddl* allele identifier onto the goeBURST results (Figure 5B and 5C). In the goeBURST tree, ST162 representing mostly penicillin susceptible isolates is surrounded by SLVs that share the same *ddl* allele (ddl14). In contrast, the SLVs of ST156 representing mostly penicillin non-susceptible isolates, show significant diversity in their *ddl* alleles. In fact, all the SLVs of these two STs that differ in the *ddl* locus are assumed by goeBURST to have originated from ST156. Further supporting the hypothesis that the variability in the *ddl* locus is a consequence of a hitchhiking effect with penicillin resistance is the fact that only 4 out of the 29 *ddl* alleles found among the SLVs are exclusively associated with penicillin non-susceptibility.

A screencast to demonstrate how fast and easy it is to produce this comparison is available at http://www.phyloviz.net/wiki/videos/.

### Remote MLST public repositories

The PHYLOViZ LoadMLST DBs plugin allows PHYLOViZ to load allelic profiles and sequence data from public MLST repositories. It is currently capable of retrieving data from five public repositories: PubMLST [5], MLST.net [4], MLST Databases at ERI [8], Institut Pasteur MLST Databases [6] and EcMLST [7]. These repositories make their data available through flat files. Additionally, this plugin allows the integration of a local file, containing the isolate ancillary data, to complement the retrieved allelic profiles.

As described in Additional file 1, each developed plugin must specify which are the necessary dependencies. In this case, the PHYLOViZ Core, PHYLOViZ MLST and PHYLOViZ Aligned Sequences need to be specified, since the plugin permits the retrieval of allelic profiles and sequence data.

The PHYLOViZ LoadMLST DBs plugin creates an entry under the File menu, to launch a wizard-like interface guiding the user through the loading process. The interface is composed of four distinct steps. The first, presents the user with the list of all the possible data sets available in the five public repositories, together with a mandatory text field where the user specifies the name of the selected data set. The second, presents the full list of genes constituting the allelic profile of the selected data set, as well as the number of STs composing it. The complete set of allelic profiles can be downloaded by a single click. In the third step, the user can integrate with the retrieved allelic profiles, a local file containing isolate ancillary data. Finally, in the last step of the wizard, the user is presented with two choices: the immediate creation of a data set without any sequence data, or to add the complete sequence data for each loci. In the latter, the user can import the sequence data independently for each locus, either by downloading it directly from the public repository or by specifying a local file in FASTA format. By pressing Finish, the new data set is created with the selected typing data.

Since both allelic profiles and sequence data are specific instances of TypingData, both the goeBURST algorithm and its MST-like approach can be directly applied. This means that PHYLOViZ permits the inference of patterns of evolutionary descent, not only from the MLST data but also directly from the sequence data of the selected data set (see Algorithms subsection).

A similar plugin (PHYLOViZ PubMLST) takes advantage of the PubMLST Web services to also automatically retrieve isolate ancillary data from the database. Both the PHYLOViZ LoadMLST DBs and the PHYLOViZ PubMLST plugins allow PHYLOViZ to directly access, analyse and visualize the latest epidemiological data available in remote MLST public repositories.

## Conclusions

PHYLOViZ provides the research community with a freely available and easy to use software tool for the analysis of sequence-based typing methods. It is in continuous development, focusing on user-friendliness, platform independence, modularity, reusability and efficiency and offering a plugin architecture gives users the possibility to create modules tailored to specific needs. Its flexibility in terms of epidemiological typing data analysis, relies on its ability to use any data format currently used for the established methods, such as MLST and MLVA, as well as those of upcoming methodologies based on NGS.

We are currently working on new analysis methods and improved visualization capabilities in order to support and efficiently analyze the continuously growing data sets. We are also extending the software to directly interact with online databases, with the ultimate goal of providing a unified and simple interface for users to interact and explore currently available data.

## Availability and requirements

∙**Project name:** PHYLOViZ ∙**Project home page:**http://www.phyloviz.net/∙**Operating system(s):** Platform independent ∙**Programming language:** Java ∙**Other requirements:** Java 1.6 or higher ∙**License:** GNU GPL v3 w/ Classpath Exception ∙**Any restrictions to use by non-academics:** Only those imposed already by the license.

## Competing interests

The authors declare that they have no competing interests.

## Authors’ contributions

APF and CV proposed the software architecture and developed PHYLOViZ. PTM evaluated the software and implemented plugins for both statistical analysis and remote data retrieval. JMC, MR and JAC proposed the project idea, evaluated the software, tested it with real data sets and analysed results, pointing out errors and improvements. All authors wrote, read and approved the final manuscript.

## Supplementary Material

Addtional file 1 PHYLOViZ. Plugin implementation example. Click here for file
