# Supplementary material for: PHYLOViZ: phylogenetic inference and data visualization for sequence based typing methods
Source: BMC Bioinformatics. 2012 May 8;13:87. doi: 10.1186/1471-2105-13-87 (PMC3403920; doi:10.1186/1471-2105-13-87)
Supplement: Addtional file 1 — PHYLOViZ. Plugin implementation example. [file 1471-2105-13-87-S1.pdf]

PHYLOViZ: Phylogenetic Inference and Data Visualization for Sequence Based Typing Methods (Supplemental Material)

Alexandre P Francisco[1,2], Cátia Vaz[1,3], Pedro T Monteiro[4,5], José Melo-Cristino[4], Mário Ramirez[4] and João A Carriço*[1,4]

[1] KDBIO, INESC-ID, R. Alves Redol 9, 1000-029 Lisboa, PT
[2] CSE Dept, IST, Tech Univ of Lisbon, Av. Rovisco Pais 1, 1049-001 Lisboa, PT
[3] DEETC, ISEL, Poly Inst of Lisbon, R. Cons. Emídio Navarro 1, 1959-007 Lisboa, PT
[4] Inst de Microbiologia, Inst de Medicina Molecular, Fac de Medicina, Univ of Lisbon, Av. Prof. Egas Moniz, 1649-028 Lisboa, PT
[5] Instituto Gulbenkian de Ciência, 2781-901 Oeiras, PT

Email: Alexandre P Francisco - aplf@ist.utl.pt; Cátia Vaz - cvaz@cc.isel.ipl.pt; Pedro T Monteiro - ptgm@igc.gulbenkian.pt; José Melo-Cristino - melo_cristino@fm.ul.pt; Mário Ramirez - ramirez@fm.ul.pt; João A Carriço*- jcarrico@fm.ul.pt;

*Corresponding author

Plugin implementation

As an example, consider the case of providing a new distance for goeBURST by making available a new plugin. For this, a developer using NetBeans should proceed as follows:

1. create a new project of type NetBeans Modules and add a new package, for instance, my.local.pkg;
2. add as module dependencies Lookup API (part of NetBenas itself), PHYLOViZ Algorithms, PHYLOViZ Core and PHYLOViZ goeBURST;
3. create a class named, for instance, NewDistanceProvider that implements the interface DistanceProvider:

```
package my.local.pkg;
import net.phyloviz.algo.AbstractDistance;
import net.phyloviz.algo.DistanceProvider;
import net.phyloviz.core.data.TypingData;
import net.phyloviz.goeburst.tree.GOeBurstNode;
import org.openide.util.lookup.ServiceProvider;
```

```
@ServiceProvider(service = DistanceProvider.class)
public class NewDistanceProvider implements DistanceProvider<GOeBurstNode> {

    public AbstractDistance<GOeBurstNode> getDistance(TypingData td) {
        return new NewDistance(td);
    }

public AbstractDistance<GOeBurstNode> getDistance(TypingData td, int maxLevel) {
        return new NewDistance(td, maxLevel);
    }

    public String toString() {
        return "New Distance";
    }
}
```

4. 4. add the missing class NewDistance that should implement the interface AbstractDistance:

```
package my.local.pkg;
import java.util.Comparator;
import net.phyloviz.algo.Edge;
import net.phyloviz.algo.AbstractDistance;
import net.phyloviz.core.data.TypingData;
import net.phyloviz.goeburst.tree.GOeBurstNode;

public class NewDistance implements AbstractDistance<GOeBurstNode> {

    private int maxLV;
    private Comparator<Edge<GOeBurstNode>> ecmp;
    private Comparator<GOeBurstNode> pcmp;
```

```
public NewDistance(TypingData td, int maxLevel) {
    maxLV = maxLevel;
    ecmp = new EdgeComparator();
    pcmp = new ProfileComparator();
}

public NewDistance(TypingData td) {
    this(td, td.getHeaders().size() - 1);
}

public int level(GOeBurstNode px, GOeBurstNode py) {
    //Add your code...
}

public int level(Edge<GOeBurstNode> e) {
    return level(e.getU(), e.getV());
}

public int maxLevel() {
    return maxLV;
}

public int compare(Edge<GOeBurstNode> f, Edge<GOeBurstNode> e) {
    return ecmp.compare(f, e);
}

public String toString() {
    return "MLVA Distance";
}

public int compare(GOeBurstNode px, GOeBurstNode py) {
```

```
    return pcmp.compare(px, py);
}

public Comparator<GOeBurstNode> getProfileComparator() {
    return pcmp;
}

public Comparator<Edge<GOeBurstNode>> getEdgeComparator() {
    return ecmp;
}

public String info(GOeBurstNode px, GOeBurstNode py) {
    // Add your code...
}

public String info(Edge<GOeBurstNode> e) {
    return info(e.getU(), e.getV());
}

private class ProfileComparator implements Comparator<GOeBurstNode> {

    public int compare(GOeBurstNode u, GOeBurstNode v) {
        return u.diffLV(v);
    }
}

private class EdgeComparator implements Comparator<Edge<GOeBurstNode>> {

    public int compare(Edge<GOeBurstNode> f, Edge<GOeBurstNode> e) {
        // Add your code...
    }
```

```
        }
    }
```

5. edit module properties and add missing information, such as module description and license;

6. package it by creating a NBM;

7. add it to PHYLOViZ.

Note that, in step 3, both implementations of the method getDistance can return null if the implemented distance is not suitable for the kind of typing data passed as parameter. This capability is of particular importance for distances that assume certain properties, such as in the case of the PHYLOViZ MLVA Distance where we require that each profile entry must be available as a double precision floating point variable. Note also that, if you are willing to use the statistics feature of goeBURST, in step 4, the absolute value of the output for the method compare of EdgeComparator will be used as the tie break level reached.
